# Chronic Rhinosinusitis and Alzheimer’s Disease—A Possible Role for the Nasal Microbiome in Causing Neurodegeneration in the Elderly

**DOI:** 10.3390/ijms222011207

**Published:** 2021-10-18

**Authors:** Sanaa Harrass, Chenju Yi, Hui Chen

**Affiliations:** 1 School of Life Sciences, University of Technology Sydney, Sydney, NSW 2007, Australia; hui.chen-1@uts.edu.au; 2 Research Centre, The Seventh Affiliated Hospital of Sun Yat-sen University, Shenzhen 518107, China; yichj@mail.sysu.edu.cn

**Keywords:** dementia, nasal microbiome, dysbiosis, inflammation, β-amyloid, upper respiratory tract

## Abstract

Among millions of sufferers of chronic rhinosinusitis (CRS), the challenge is not only constantly coping with CRS-related symptoms, such as congested nose, sinus pain, and headaches, but also various complications, such as attention difficulties and possible depression. These complications suggest that neural activity in the central nervous system may be altered in those patients, leading to unexpected conditions, such as neurodegeneration in elderly patients. Recently, some studies linked the presence of CRS and cognitive impairments that could further develop into Alzheimer’s disease (AD). AD is the leading cause of dementia in the elderly and is characterised by progressive memory loss, cognitive behavioural deficits, and significant personality changes. The microbiome, especially those in the gut, has been recognised as a human organ and plays an important role in the development of various conditions, including AD. However, less attention has been paid to the microbiome in the nasal cavity. Increased nasal inflammatory responses due to CRS may be an initial event that changes local microbiome homeostasis, which may further affect neuronal integrity in the central nervous system resulting in AD. Evidence suggests a potential of β-amyloid deposition starting in olfactory neurons, which is then expanded from the nasal cavity to the central nervous system. In this paper, we reviewed currently available evidence that suggests this potential mechanism to advise the need to investigate the link between these two conditions.

## 1. Introduction

Recently, the microbiome has been recognised as a human organ [1]. The altered gut microbiome has been well documented in several mental disorders and neurodegenerative disorders, and its potential as a therapeutic strategy has also been proposed in such conditions [2,3,4,5,6]. Similarly, the nasal cavity is a habitat for a diverse microbial community, which can also play an important role in human health. To date, the research in this area mainly focuses on conditions within the respiratory system, such as asthma and cystic fibrosis [7,8]. However, it also needs to be noted that the nasal cavity constitutes a very important route of entry for pathogens that can directly spread into the central nervous system, which may initiate or worsen neurodegenerative disorders, such as Alzheimer’s disease (AD) [9]. Nonetheless, insufficient attention has been given to the potential association between AD and chronic rhinosinusitis (CRS), a chronic condition of the nasal cavity that can alter the homeostasis of the local microbiome.

Chronic inflammation is involved in many conditions, including CRS and AD, which may bridge the presence of CRS and the risk of dementia [9,10]. In addition, CRS caused dysbiosis of the nasal microbiome can contribute to the development of AD through several mechanisms, including modulation of immune response in the central nervous system and/or direct translocation of bacteria or bacterial byproducts to the central nervous system to initiate inflammation. This hypothesis is further supported by improved cognitive performance in those with effective CRS treatments [11,12]. In this review, we will go through currently limited studies to demonstrate the need for more research on the potential causative effect of CRS on AD development.

## 2. Nasal Microbiota

The nasal cavity is exposed to vast amounts of bacteria and viruses during breathing ~7000 L of air each day. The inhaled air can be loaded with different viruses and bacteria [13]. The physical function of the nasal epithelium is to form a barrier between the internal and external environments (7), by detecting and eliminating pathogens to prevent them from initiating infection in underlying cells locally and reaching the lower respiratory tract [14]. This is why the nasal epithelium has innate immune defences, such as lysozyme, lactoferrin, IgM and IgA, to fight against these pathogens [15]. However, the nasal cavity is also a house for resident microbial communities that play an important role in maintaining a healthy environment and prevent infection and inflammation [16]. For example, for opportunistic pathogens, nasal commensal bacteria can inhibit the infection and further spreading of such pathogens by depriving them of space and nutrients, as well as actively secreting toxic chemicals to prevent their thriving; on the other hand, dysbiosis can increase the susceptibility to certain external pathogen infections, e.g., influenza [17]. Even for the current pandemic pathogen SARS-CoV-2 that primarily enters the human body via the nasal cavity that is also the first site to get infected, it is suggested that the local response of the nasal commensal bacteria may affect local nasal mucosal barrier integrity for bacterial entry into the circulation, and regulate systemic immune response (e.g., activation of T regulatory cells and myeloid-derived suppressor cells) and subsequent disease severity [17]. Although there has been no successful clinical trial targeting nasal bacteria for disease treatment, healthy commensal bacterial compositions are important in preventing airborne bacterial and viral infections.

The nasal microbiome, like other body microbial niches, develops throughout the human life span. Before birth, foetuses are developed in a sterile uterus. During birth, the newborn gets the first contact with microorganisms from the vaginal canal during natural birth, or through skin contact in the case of caesarean section [18]. The nasal and nasopharyngeal microbiota start to shape after birth [19]. Several factors play a vital role in shaping the early microbiome, such as breastfeeding and respiration [20]. However, the diversity of neonatal microbiota remains low at birth [21]. This bacterial diversity increases over the first few months until the age of three. Afterwards, bacteria in the upper respiratory tract become more stable and resemble that of adults [22].

The nasal cavity has a diverse microbial community. The healthy nasal cavity is colonised with *Actinobacteria*, *Bacteroidetes*, *Firmicutes*, and *Proteobacteria* on the phylum level, and *Bifidobacterium*, *Corynebacterium*, *Staphylococcus*, *Streptococcus*, *Dolosigranulum* and *Moraxella* on the genus level [23,24]. The nasal cavity may also harbour some pathogenic bacteria in a healthy status, such as *Staphylococcus aureus* and *Hemophilus influenza* [25]. However, these are opportunistic bacteria, therefore, only cause significant illness if a person becomes immunocompromised [26].

Similar to the intestine, changes in nasal microbiome homeostasis may play a significant role in disease progressions, such as CRS, allergic rhinitis, and asthma [27]. This dysbiosis is characterised by a reduced population of beneficial bacteria and the overgrowth of pathogens. Bacterial dysbiosis may start at early infancy or develop later in life. For example, Teo et al. observed nasopharyngeal bacteria of infants in their first year and showed that certain bacterial composition in the nasopharynx is a predictor of future development of asthma in these infants, with *Streptococcus* species specifically the main contributor to this outcome [28]. This study highlighted the importance of the nasal microbiome composition in infants as an indicator of future chronic pulmonary inflammatory disease. Another case-control study highlighted the role of the nasal microbiota in early life in the development of allergies in the upper respiratory tract in infants [29]. Nasal microbiota diversity is increased with age in healthy children, whereas diversity is decreased with age in children with rhinitis [29]. Thus, the nasal microbiome may play an important role in developing inflammatory diseases in the respiratory tract.

Moreover, aging plays a critical role in shifting the nasal microbiome in health and disease. In adulthood, the microbiota of the nasal cavity is distinct from the microbial community in other parts of the upper respiratory tract; the microbial composition remains relatively constant throughout adulthood [30]. However, alteration in the nasal microbiota has been observed in middle-aged individuals. In healthy adults aged 40–65 years, the microbiota is altered and dominated by *Staphylococcus*, *Cutibacterium* and *Corynebacterium* [31]. The bacterial composition changes again in people aged 65 years and over, and is dominated by oropharyngeal bacteria [32,33]. The spread of bacteria from the distinct niche of the oropharynx upwards to the nasopharyngeal region can be due to the weakening of the immune system with aging (immunosenescence), leading to increased pro-inflammatory markers, lowered ability to manage immune stress, and the loss of bacterial niches and decreased bacterial diversity [32].

## 3. CRS, Nasal Microbiota and Their Influence on Neurological Health

**Figure 1:**
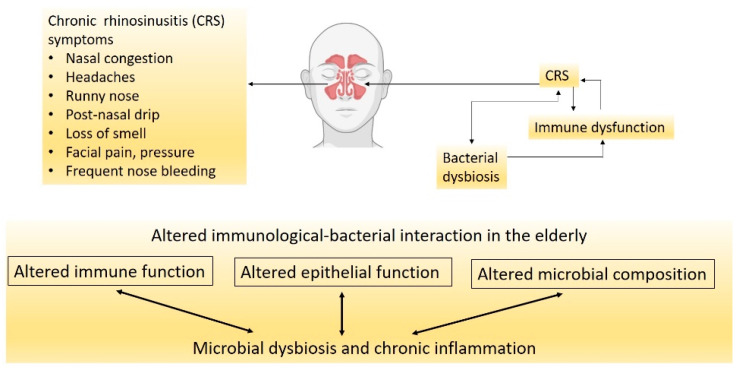
CRS is a multifactorial disease with the exact triggers not fully understood. Factors that cause bacterial dysbiosis could worsen with age by promoting immunological dysfunction.

CRS is a chronic inflammatory disease of the nasal cavity and sinuses that lasts for more than three months [34]. CRS is a debilitating disease that negatively affects life quality and poses an economic burden on the community. The disease is characterised by persistent inflammation of the nasal cavity and paranasal sinuses that results in symptoms of nasal obstruction, rhinorrhea, facial pain, headache and loss of smell (Figure 1) [35]. CRS is affected by three major factors: altered epithelial barrier and immunity, chronic inflammation, and nasal microbial dysbiosis (Figure 1) [36].

Although limited studies are available, lower nasal microbial diversity has been found in patients with CRS [37]. At the phylum level, there is a decrease in *Actinobacteria* and *Firmicutes* and an increase in *Proteobacteria* numbers [38]. In terms of the species, there is an increase in opportunistic pathogenic bacteria, such as *S. aureus* and *Corynebacterium tuberculosteriaticum* [38]. Although only a few studies have investigated the nasal microbiome in the elderly with CRS, some evidence suggests immunological mechanisms leading to bacterial dysbiosis in this age group. Epithelial cell integrity and regeneration are impaired in adults with CRS and even to a greater extent in the elderly. For example, S100 family proteins involved in epithelial proliferation, repair and defence against pathogens are reduced with aging [39]. Moreover, the epithelial changes are involved in reduced mucociliary clearance in the healthy population aged 40 years and over, along with microtubules disarrangement [40,41]. Another study showed that mucociliary clearance has been significantly diminished in people older than 60 years with diabetes and hypertension, independent of smoking [42]. Similarly, a mouse study showed that mucociliary clearance was diminished in the upper and lower airways of elderly mice compared with young mice [43]. Moreover, elderly people with upper respiratory tract allergies have also presented with thinner nasal mucosa, which can be due to reduced blood flow to the nasal cavity and reduced mucus production [44]. Biopsy samples from the elderly also showed reduced thickness of the epithelium and basement membrane resulting in increased volume of the nasal cavity [45]. All these changes in the epithelium with aging can lead to less pathogen clearance, nasal dysbiosis and hence, the translocation of oropharyngeal microbiota to the nasal cavity.

In addition to physiological changes, immune responses are also altered in the elderly. For example, Cho et al. observed an age-dependent loss of immune function in the upper respiratory tract of CRS patients [39]. They showed that the eosinophilic inflammatory marker in the nasal cavity was significantly lower in CRS patients aged 60 years and over compared to that in younger patients, although eosinophilic infiltration was the same between the two groups [39]. The authors attributed this feature to eosinophils being less active in the older group. This suggests that there is an age-dependent loss in the function of the immune system in CRS patients. Thus, impaired immune response to new and probably existing pathogens in the elderly may be an important factor in their higher susceptibility to infections, the persistence of CRS, and probably the development of other inflammatory diseases in this population.

The interaction between the epithelium and bacterial residents may be a determinant of homeostasis or local inflammation. The sinonasal epithelium contains complex innate and adaptive immune pathways that drive inflammatory responses to pathogens in order to protect the host from exogenous or resident pathogen infections [46]. Any inappropriate activation or lack of inhibition of the immune system can lead to chronic inflammation [47]. Because bacterial pathogens are often observed in CRS, it is speculated that bacterial dysbiosis plays a vital role in initiating or contributing to chronic nasal inflammation [10]. Due to the proximity of the nasal cavity to the brain, inflammatory diseases in the nasal cavity, such as acute and chronic sinusitis, can initiate a wide range of neurological complications, including epidural abscess, meningitis, brain abscess, venous sinus thrombosis and orbital cellulitis [48]. These neural infections have common consequences, such as permanent visual changes and epilepsy [48]. The nasal-induced neural infections in the central nervous system suggest that the inflammatory environment in the nasal cavity can affect the brain. Hence, it is hypothesised that the inflammatory milieu in the nasal cavity can also lead to the initiation and/or the development of certain neurodegenerative diseases, such as AD.

## 4. Alzheimer’s Disease

AD is a neurodegenerative disorder that leads to memory loss and cognitive impairments. It is the most common cause of dementia, with the risk doubling every five years in the elderly population [49]. It is a global public health concern rising exponentially, with around 50 million individuals suffering worldwide due to the increase in the aging population and lack of effective treatments [50]. The early sign of this disease is mild cognitive impairment (e.g., short term memory loss). As the disease progresses, more severe neurological impairments appear, such as severe memory loss, cognitive behavioural and personality changes, difficulties in performing everyday tasks and loss of communicating capability [51]. The disease eventually progresses into significant changes in personality and behaviour, impaired immune function, extreme difficulties in comprehension and communication and problems with movement and swallowing [52,53].

AD is a multifactorial disease with several well-accepted risk factors, such as old age, genetic predisposition, cardiovascular and cerebrovascular diseases, environmental factors and infections [54,55,56,57]. The risk of this disease is higher in women; more than 65% of late-onset AD cases are women [58]. This sex difference in AD risk is partially due to the fact that women are more likely to carry an apolipoprotein (E ε4 allele) correlated to AD [59]. Moreover, exposure to ovarian hormones can also play a role in sex differences in AD risk, with more total reproductive years in women contributing to lower AD risk and vice versa [60].

The widely-accepted pathological mechanisms of AD include β-amyloid (Aβ) aggregations, tau hyperphosphorylation-induced neurofibrillary tangles, inflammation due to abnormal microglial function, synapse loss due to microglial and astrocyte dysfunction, pericyte dysfunction, and mutation in the apolipoprotein E gene [61,62,63]. Aβ deposition and neurofibrillary tangles are the hallmarks of pathological changes in AD brains. Aβ is derived from amyloid protein precursor and was originally suggested, particularly Aβ1-42, to be the main trigger of neuropathology in AD [64]. Nowadays, studies on AD still place Aβ as an important target for investigation; however, the failure to develop a drug has undermined the Aβ-based therapeutic approaches [65,66]. Lately, Aβ clearance rather than synthesis has been recognised to play a more important role in Aβ aggregation. In healthy brains, the clearance of Aβ is higher than its synthesis, and hence accumulation is unlikely to happen [67]. On the other hand, the defects in Aβ clearance lead to extracellular plaque aggregates that are highly toxic to neurons and cause synaptic dysfunction and inflammation, as well as astrogliosis and microgliosis that secrete cytotoxic substances, which eventually lead to neuronal damage, atrophy and death [68,69]. In addition to Aβ, tau (tubulin-associated unit) protein pathology adds to the disease burden. Tau protein is primarily expressed in the axons of the central nervous system, and hyperphosphorylation of this protein causes the formation of neurofibrillary tangles inside neurons, contributing to AD pathology [70]. Despite three decades of research, the pathogenesis of AD is still not well understood, and there is no effective treatment to slow down or stop its progression.

## 5. CRS and AD—How Close Are They?

Researchers have investigated the correlation between dementia and CRS. A recent retrospective study following patients from 2006 to 2019 found that in patients with CRS, the risk of dementia was also increased [71]. This study followed patients with mild cognitive impairment with or without CRS for the study period, which found that patients with mild cognitive impairment and CRS were more likely to develop dementia than those with mild cognitive impairment but without CRS [71]. Similarly, a case-control Taiwanese study included 8768 dementia patients and confirmed significantly higher odds of CRS in the dementia population [72]. The authors suggested that CRS is associated with other comorbidities, such as stroke and vasculopathy, leading to an increased risk of vascular dementia. In addition, several other studies have also found a correlation between CRS and cognitive impairment [73,74,75].

Some studies, however, failed to find a correlation between AD and CRS [76]. This may be due to the variation in how CRS was diagnosed. In some cases, CRS was self-diagnosed, and even with clinical examination, it is hard to differentiate between CRS and rhinitis. Thus, computed tomography (CT) scans are needed to confirm the diagnosis, which is not routinely performed in every patient [77]. Another reason why studies failed to find a correlation between CRS and dementia is that dementia is a disease manifested in old age, and CRS symptoms can be improved with aging due to the alterations in the immune system. For example, Holmes et al. showed that the burden of CRS is higher in patients under 39 years of age than elderly patients [78]. Another study found that in CRS patients, the nasal epithelial barrier function is worsened with aging [39]. This reduced inflammatory burden of CRS in the aging population may make it harder to be recognised by the patients themselves, and subsequently identified and diagnosed by the clinicians.

Even with impaired epithelial integrity in elderly patients, a bacterial infection in the sinuses cannot spread to the brain because of the blood-brain barrier; however, the inflammation can spread through the olfactory bulb and the olfactory neural system to reach the brain where the blood-brain barrier is lacking. Chronic inflammation is present in both CRS and AD, which may bridge CRS and the risk of neurodegeneration causing dementia [79]. It is well known that increased brain inflammation causes cognitive decline even before the onset of AD neuropathology, i.e., Aβ aggregation and tau hyperphosphorylation [80]. Inflammatory cytokines from active microglia and astrocytes impair cortical function and reduce hippocampal volume, which leads to memory and learning impairments [81]. Moreover, human brains with Aβ aggregations have microglial activation and increased pro-inflammatory cytokine production; increased circulating levels of inflammatory proteins, such as C-reactive protein, are also correlated with the presence of dementia [82,83]. During the early stages of AD, microglia and astrocytes are activated and able to clear Aβ, but the chronic activation of those cells has detrimental effects due to the secretion of inflammatory mediators tumour necrosis factor-α (TNF-α) and interleukin (IL)-6 [84]. These inflammatory mediators play crucial roles in the neurodegenerative process due to less Aβ clearance and increased accumulation. Interestingly, the role of inflammation in AD has been supported by a human study showing that AD risk was lowered by anti-inflammatory medications [85].

**Figure 2:**
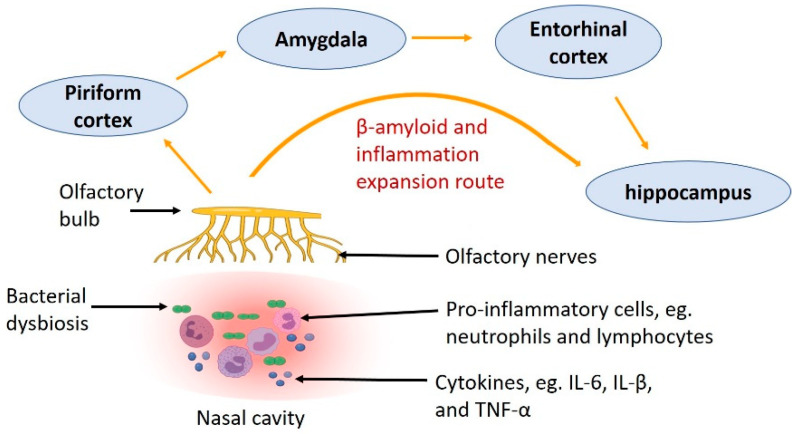
The route showing how inflammation caused by nasal bacterial dysbiosis and Aβ deposition in the olfactory nerve in CRS spread to other parts of the central nervous system, through the olfactory bulb to the piriform cortex, amygdala, entorhinal cortex and hippocampus.

In CRS, the immune system is dysregulated, which may be the driving force for inflammation. The innate immune system is suppressed with decreased immunoglobulin J chain, antileukoproteinase, tertiary lymphoid structure and surfactant protein-A [86]. On the other hand, immune cells, such as eosinophils and basophils, are increased in patients with CRS [87]. Activation of these inflammatory cells leads to the recruitment of more cells, the polarisation of Th2 cells, and the production of inflammatory cytokines, such as IL-13, IL-5 and IL-4 [88]. Moreover, inflammation disrupts nasal epithelial cell regeneration through the inhibition of neural progenitor cell proliferation, which may aggravate CRS [89]. Such inflammatory response due to CRS can further affect the development of AD (Figure 2). A meta-analysis shows that dementia is associated with increased circulating inflammatory mediators, e.g., IL-6, IL-12, IL-18, TNF-α, IL-1β and transforming growth factor-β (TGF-β) [90]. IL-1β, IL-6, TNF-α and TGF-β are also elevated in the mucosa of patients with CRS [91,92,93,94]. However, it is unclear if increased cytokines in the nasal cavity can directly affect neural integrity in the central nervous system that leads to neurodegeneration.

## 6. Expansion Route of Aβ—From the Nose to the Brain

There are some studies suggesting the possibility of Aβ deposition expansion from the olfactory neurons in the nasal cavity to the central nervous system causing the development of AD. In AD patients, brain regions close to the olfactory area (such as the hippocampus and entorhinal cortex), which lies in the roof of the nasal cavity, showed significant neuropathology and dysfunction, including the loss of neurons and atrophy [95,96,97,98]. Furthermore, another study using a mouse model of AD (AβPP/PS1 transgenic mice) found that Aβ in the olfactory epithelium spreads to the olfactory bulb, followed by the anterior olfactory nucleus, piriform cortex, entorhinal cortex and hippocampus [99]. Such Aβ aggregates in the abovementioned brain regions are increased with age [99]. These studies demonstrate that Aβ can spread from the nasal cavity to central brain areas in an age-dependent manner (Figure 2).

Other brain regions affected during the early stage of AD are the areas from the olfactory bulb to the olfactory tract and the olfactory cortical area, which is anatomically close to the hippocampus [100]. The olfactory bulb, anterior olfactory nucleus and olfactory cortex are present with neurofibrillary tangles in the early stages of AD before the pathology appears in the other parts of the brain remote to olfactory-related regions [101]. Some recent evidence confirmed that Aβ and Tau proteins can be propagated through synapses [102]. The olfactory projection neurons innervate multiple cortical regions, and the mitral and tufted cells have different projections to different brain regions. The mitral cells project to the olfactory nuclei, the olfactory tubercle, the entorhinal cortex and portions of the amygdala. The pyramidal cells project into the thalamic and hypothalamic nuclei, the hippocampus and amygdala [103]. These projections of the olfactory bulb to central brain areas suggest that the initial event of Aβ aggregation in the nasal cavity may contribute to the initiation of AD pathology in remote neurons through transmitting pathological protein from olfactory to cortical regions [70].

In addition, Yoo et al. identified Aβ in the nasal discharge of AD patients [104]. They also found higher levels of Aβ from the nasal discharge in individuals with high AD risks, reflected by lower Mini-Mental State Exam scores (indicating poor cognitive functions), higher Clinical Dementia Rating, and higher Global Deterioration Scale compared to healthy controls [104]. This suggests that Aβ in the nasal cavity may be an early diagnostic marker for AD and olfactory neuropathy can potentially be a risk factor for AD pathogenesis.

## 7. Potential Mechanisms Linking Nasal Microbiota and AD

CRS and aging may act synergically to amplify the damage of the nasal neuroepithelium with decreased repairing mechanisms due to aging [105,106]. As a consequence, the inflammatory cells in the nasal cavity and/or the dysbiotic-nasal microbiome are able to translocate to the central nervous system to cause AD. To date, there is no direct evidence to support the initiation of AD-like neuronal pathology by the inflammatory response in the nasal cavity due to bacterial infection. However, cognitive dysfunction has been found to be attenuated after sinus therapies or surgery. In a prospective study, treatment for CRS also improved cognitive dysfunction in CRS patients [12]. A multicenter study also found that CRS patients showed improved cognitive scores after undergoing sinus surgery [107]. This improvement in cognitive function can be attributed to reduced local inflammation or improved oxygen supply after the therapy or surgery that positively impact neighbouring cells in the brain. As such, further study may examine the nasal cavity from postmortem AD patients at all stages to support or rule out the hypothesis of the nasal origin of AD.

The nasal microbiota of AD patients has not been thoroughly examined; however, there is some evidence suggesting that the normal population is disrupted with an increased number of pathogens. For example, pneumonia caused by *Chlamydia pneumonia* (*C. pneumonia*), is the most common cause of death in AD patients and has also been suggested as one of the pathogens that can initiate the development of AD [108,109]. *C. pneumonia* is an obligate intracellular pathogen that infects humans and is thought to be responsible for 5% of sinusitis cases and 15% of community-acquired pneumonia cases [110]. A postmortem study found *C. pneumonia* in the olfactory bulb of 89% of AD brains compared to 5% in the controls, independent of pneumonia at the time of death [111]. *C. pneumonia* has also been found in the astrocytes, microglia and neurons of these AD brains [111]. Astrocytes play an important role in the central nervous system, such as defence against oxidative stress, fluid and electrolyte homeostasis, tissue repair and energy metabolism [112]. Microglia plays a curtail role as the immune cells of the brain and are responsible for fighting infections and inflammation [113]. During brain infection, astrocytes and microglia react to invading pathogenic bacteria by producing reactive oxygen species and pro-inflammatory cytokines to eliminate the pathogens (such as *C. pneumonia* that is correlated with AD pathogenesis) [114]. *C. pneumonia* was found viable in the astrocytes and microglia from AD brains and these glial cells were close to neurofibrillary tangles and senile plaques in the AD brains [114]. These findings suggest the possible role of respiratory pathogens in the pathogenesis of AD.

In addition, post mortem studies showed that AD brains have a 5- to 10-fold increase in bacterial load over the controls, including species associated with oral, nasopharyngeal and skin niches, such as *P. acnes* [115]. Moreover, bacterial endotoxin lipopolysaccharides were also found in the affected AD brain regions, with 21-fold increase in the hippocampus and 7-fold increase in the neocortex compared to the controls [116]. Moreover, bacterial toxins, such as diphtheria toxin produced by *Corynebacterium diphtheria*, a bacteria commonly found in the nasopharynx, is hypothesised to be able to ascend the central nervous system from the nasopharynx to cause sporadic AD which starts from the entorhinal cortex and develops into the hippocampus and other neocortical areas [117].

If there is a correlation between CRS and dementia as hypothesised above, CRS treatments should, in theory, be able to ameliorate neurodegeneration and improve, preserve or delay cognitive functional decline. Currently, CRS treatments include medication and surgical options by addressing the infection and reducing sinonasal inflammation. The medications are a combination of antibiotics, nasal decongestants, steroids and saline irrigation [118]. Acute bacterial sinusitis is treated successfully with antibiotics, but a combination of antibiotics and corticosteroids is used for CRS [119]. Aminoglycosides are a class of antibiotics historically used as nasal irrigations or sprays to reduce symptoms and bacterial burden in CRS [120]. A recent study found that the aminoglycosides were able to rescue a mutation that is prevalent in some frontotemporal dementia patients [121]. This mutation prevents the neuronal cells from making a protein called progranulin, the absence of which is linked to frontotemporal dementia. Although the function of progranulin is not fully understood, this discovery is promising for drug development for dementia [121].

## 8. Future Research

Studies identifying the changes in the nasal microbiome profile of the elderly are limited, especially in those with significant cognitive functional declines. The potential involvement of CRS in the aging population, e.g., those above 60 years old, warrants further investigation. The dysbiosis and chronic low-grade inflammation in the nasal cavity can contribute to the propagation of the local inflammation into proximal brain regions. Thus, prospective cohort studies are needed to investigate the association between AD and CRS.
